# Transcriptomic dataset for Sardina pilchardus: Assembly, annotation, and expression of nine tissues

**DOI:** 10.1016/j.dib.2021.107583

**Published:** 2021-11-17

**Authors:** Jorge Langa, Martin Huret, Iratxe Montes, Darrell Conklin, Andone Estonba

**Affiliations:** a Department of Genetics, Physical Anthropology and Animal Physiology, Faculty of Science and Technology, University of the Basque Country, UPV/EHU, Leioa, Bizkaia 48940, Spain; b IFREMER, STH/LBH, B.P. 70, Plouzané 29280 France; c Department of Computer Science and Artificial Intelligence, Faculty of Computer Science, University of the Basque Country UPV/EHU, San Sebastián, Spain; d IKERBASQUE, Basque Foundation for Science, Bilbao, Spain

**Keywords:** *Sardina pilchardus*, European sardine, Transcriptome assembly, Annotation, Expression, Tissue quantification, Pathway, Gene ontology

## Abstract

European sardine or pilchard is a planktonic small pelagic fish present from the North Sea in Europe to the coast of Senegal in the North of Africa, and across the Mediterranean sea to the Black Sea. Ecologically, sardines are an intermediary link in the trophic network, preying on plankton and being predated by larger fishes, marine mammals, and seabirds. This species is of great nutritional and economic value as a cheap but rich source of protein and fat. It is either consumed directly by humans or fed as fishmeal for aquaculture and farm animals. Despite its importance in the food basket, little is known about the molecular mechanisms involved in protein and lipid synthesis in this species. We collected nine tissues of *Sardina pilchardus* and reconstructed the transcriptome. In all, 198,597 transcripts were obtained, from which 68,031 are protein-coding. Quality assessment of the transcriptome was performed by back-mapping reads to the transcriptome and by searching for Single Copy Orthologs. Additionally, Gene Ontology and KEGG annotations were retrieved for most of the protein-coding genes. Finally, each library was quantified in terms of Transcripts per Million to disclose their expression patterns.

**Specifications Table**

**Table:** 

Subject	Omics: Transcriptomics
Specific subject area	Transcriptomics, Genomics, Fisheries, Aquaculture
Type of data	Tables, Figures, FASTA Assembly, FASTQ read files
How data were acquired	Illumina HiSeq 2000 sequencing platform
Data format	Raw reads(FASTQ) Assembly (FASTA) Annotation (TSV) Quantification (TSV)
Parameters for data collection	Three sardines were collected by IFREMER during a scientific bottom trawl survey.
Description of data collection	Total RNA was collected from nine tissues: brain, eye, heart, kidney, liver, muscle, ovary, skin, and testes. Sequencing was performed using an Illumina HiSeq 2000, yielding single-stranded paired-end reads with a length of 101 bp. Reads were cleaned with Trimmomatic. Assembly was performed with Trinity. Assembly quality was assessed with Bowtie2 and BUSCO. Annotation was done with TransDecoder and Trinotate. Quantification was performed with kallisto and sleuth.
Data source location	IFREMER survey EVHOE 2015, 31-10-2015, Bay of Biscay, 47°18′ N, 2°46′ W
Data accessibility	Raw RNA-seq reads of *Sardina pilchardus* are deposited at ENA Bioproject PRJEB18441 https://www.ebi.ac.uk/ena/browser/view/PRJEB18441. The following tissues are available: brain (ERR5925802; https://www.ebi.ac.uk/ena/browser/view/ERR5925802), eye (ERR5925802; https://www.ebi.ac.uk/ena/browser/view/ERR5925803), heart (ERR5925802; https://www.ebi.ac.uk/ena/browser/view/ERR5925804), kidney (ERR5925802; https://www.ebi.ac.uk/ena/browser/view/ERR5925805), liver (ERR5925802; https://www.ebi.ac.uk/ena/browser/view/ERR5925806), muscle (ERR5925802; https://www.ebi.ac.uk/ena/browser/view/ERR5925807), ovary 1 (ERR5925802; https://www.ebi.ac.uk/ena/browser/view/ERR5925808), ovary 2 (ERR5925802; https://www.ebi.ac.uk/ena/browser/view/ERR5925809), skin (ERR5925802; https://www.ebi.ac.uk/ena/browser/view/ERR5925810), and testes (ERR5925802; https://www.ebi.ac.uk/ena/browser/view/ERR5925811) Supplementary data is available at Figshare under DOI 10.6084/m9.figshare.14617149 (https://doi.org/10.6084/m9.figshare.14617149.v1) .

**Value of the Data**

• We present the Illumina sequencing effort and *de novo* transcriptome assembly of *Sardina pilchardus*, an important small pelagic fish due to its nutritional, economic, and ecological value.
• This data will facilitate genome annotation and the discovery of genes of interest for the aquaculture industry. This resource could serve as the basis of a SNP chip that could differentiate the stocks of sardines across the Atlantic Ocean and the Mediterranean Sea.
• The transcriptome, annotation, and expression patterns can be used to study the genes and pathways involved in ω-3 fatty acid synthesis and storage.
• The tissue quantification can be used to perform an RT-qPCR of a transcript of interest, using the tissue in which we know the target gene is active.
• Comparative evolutionary studies can be done to unravel the phylogenetic relationship of the sardine within the Clupeiformes or other teleost species.
• Selection signatures can be identified by investigating functional differences between orthologous genes in sardines and other Clupeiformes species inhabiting different environments.

## 1 Data Description

**Fig. 1:**
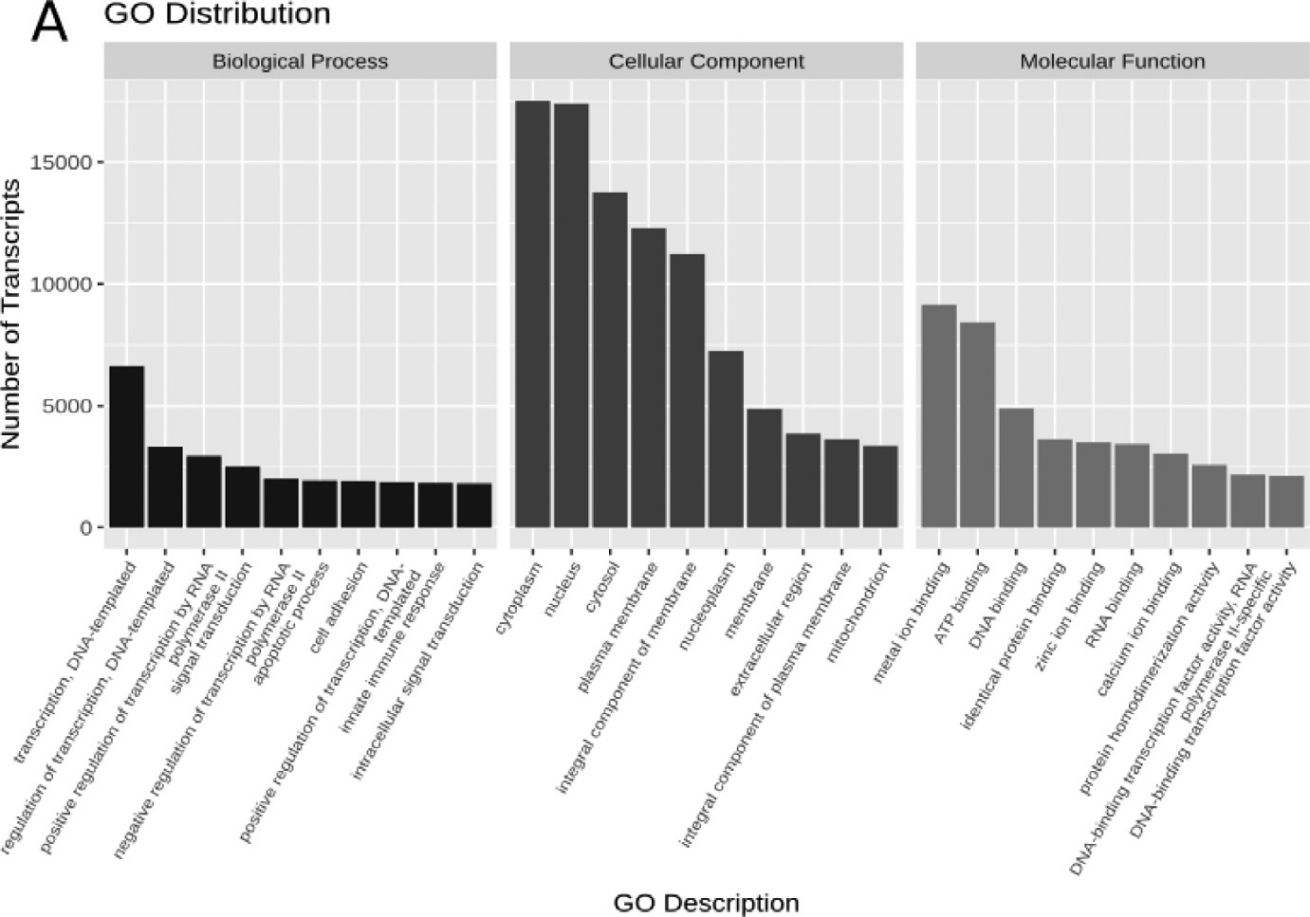

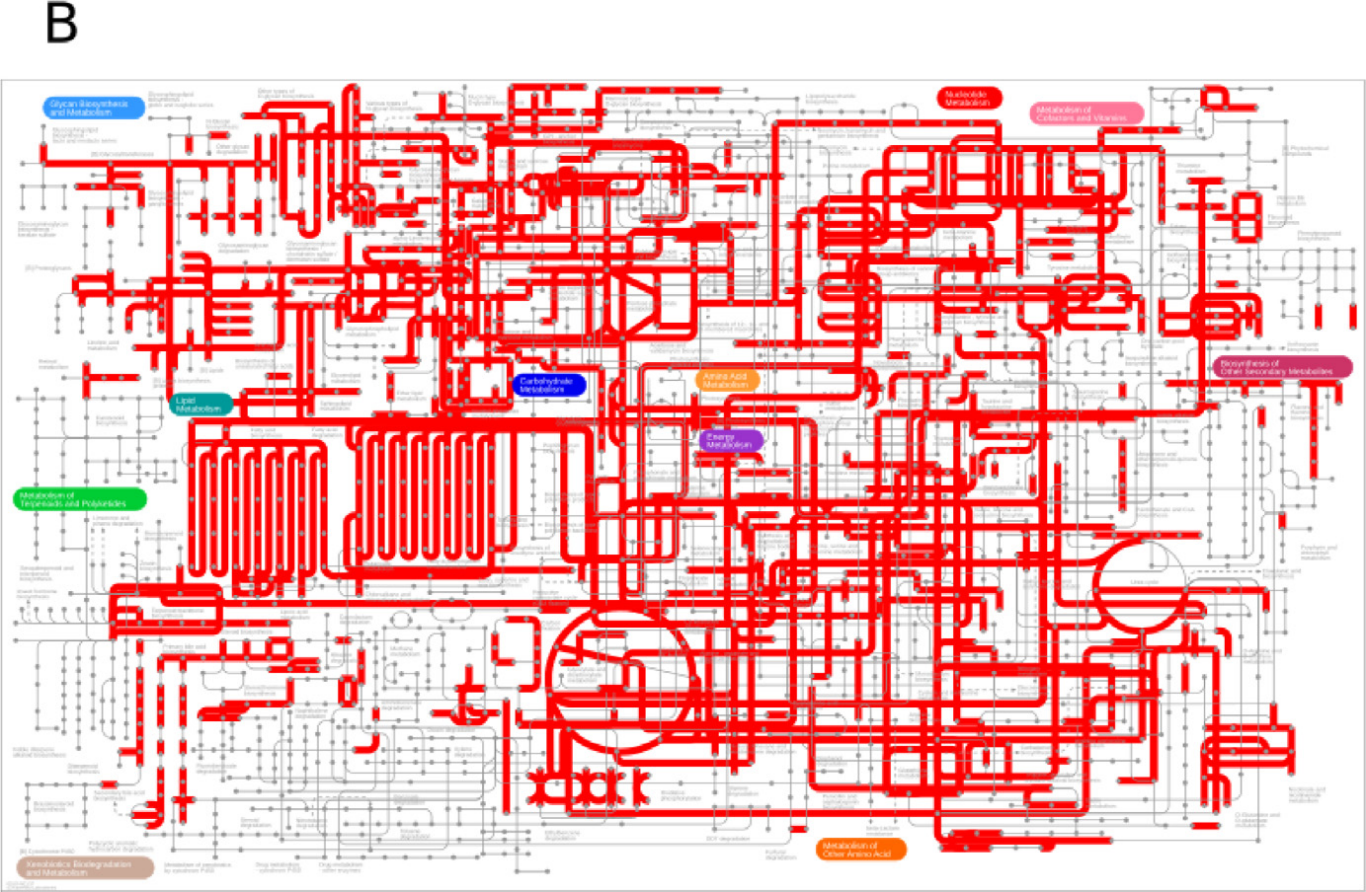
A. Gene Ontology annotation of the *Sardina pilchardus* transcriptome. The figure shows the top ten level 2 categories within the three principal categories. B. Expressed metabolome of *Sardina pilchardus* based on the KEGG annotation.

This dataset contains the RNA-Seq analysis of nine tissues of *Sardina pilchardus*. Nine tissues from two female and one male sardines were dissected onboard and immersed immediately in RNAlater. Sequencing was performed using the Illumina HiSeq 2000 platform, yielding 56 million single-stranded paired-end reads of length 101 base pairs, a median quality value per sequence of 37, 5.6 million reads per sample on average, resulting in a total of 5.70 Gbp (Table 1). Reads were preprocessed with Trimmomatic, which slightly reduced the dataset to 98,09% of the reads, and the mean read length to 100.67 base pairs. Clean reads were assembled with Trinity. To measure the quality of the assembly, cleaned reads were back-mapped to the reference, and transcripts were searched for *Actinopterygii* Single-Copy Orthologs (SCOs). Transcripts were annotated with TransDecoder and Trinotate. Results of the sequencing effort and read cleaning are available in Table 1, while the ones of assembly, quality control and annotation are in Table 2. Fig. 1 shows the most frequent Gene Ontology annotations received, and the coverage of the metabolome based on the KEGG annotations. Finally, each library was quantified with kallisto and prepared for differential downstream analysis with sleuth to obtain the expression patterns for each transcript in every tissue. The raw reads for the nine tissues of *Sardina pilchardus* have been deposited at the European Nucleotide Archive, under the umbrella project PRJEB18441, while each experimental run is deposited under accession numbers ERR5925802 to ERR5925811 (Table 1). To our knowledge, this is one of the widest datasets not only in Clupeiformes but also in fish in general, only surpassed by the ones in [1]. Supplementary data with the raw transcriptome assembly, predicted protein-coding sequences, transcript annotation and tissue quantification are available at Figshare under DOI 10.6084/m9.figshare.14617149. It includes: the assembled transcriptome (sd01-assembly.fasta), the predicted coding-sequences (sd02-transdecoder.cds), annotation (sd03-trinotate.tsv) and expression profiles per tissue (sd04-tpms.tsv).

**Table 1:** Summary of the read cleaning and backmapping of every library against the assembled reference.

Library	Sample	Accession number	Raw reads (*M*)	Trimmed reads (*M*)	Trimmed %	Trimmed Gbp	Mapped %
Brain	F1	ERR5925802	6,11	6,00	98,29	0,60	95,88
Eye	F1	ERR5925803	5,34	5,23	97,99	0,53	98,38
Heart	F1	ERR5925804	4,98	4,89	98,24	0,49	98,99
Kidney	M	ERR5925805	6,68	6,56	98,18	0,66	97,20
Liver	F1	ERR5925806	4,67	4,59	98,23	0,46	98,86
Muscle	F1	ERR5925807	5,31	5,24	98,67	0,53	98,21
Ovary 1	F1	ERR5925808	6,64	6,50	98,00	0,66	98,03
Ovary 2	F2	ERR5925809	6,57	6,41	97,60	0,65	98,05
Skin	M	ERR5925810	5,17	5,06	97,90	0,51	97,46
Testes	M	ERR5925811	5,04	4,93	97,84	0,50	97,09
Total			56,52	55,43	98,09	5,58	97,77

Sample: sample used, M for male, F1 and F2 for the females.

Raw: Original number of reads from the sequencer, in millions.

Clean: number of reads free of adapters and sequencing errors, in millions.

Clean %: Fraction of the original reads free of adapters and sequencing errors.

Clean Gbp: Total number of error-free bases, in giga base pairs.

Mapped %: Fraction of the trimmed reads that are back-mapped to the transcriptome.

**Table 2:** Summary statistics of *de novo* transcriptome assembly, quality assessment, and annotation for *Sardina pilchardus* using nine tissues.

**Assembly description**	
Assembled transcripts	198,597
Unigenes	149,981
Assembly length (Mbp)	149.36
N10	3475
N30	2080
N50	1280
Average contig length	752.08
Longest contig length	10,795
GC%	48,1
**Quality Control**	
Mapped reads	97,80%
*Actinopterygii* BUSCOs	4584
Complete, single copy	45,60%
Complete, duplicated	27,80%
Fragmented	11,60%
Missing	15,00%
**Annotation**	
Predicted ORF	68,031
Complete proteins	24,187
Contigs with match to SwissProt	67,772
Contigs with GO term	66,396
Contigs with PFAM domain	45,154
Contigs with KEGG annotation	59,254

## 2 Experimental Design, Materials and Methods

### 2.1 Sampling strategy

Three individuals from the European Atlantic Ocean were collected by the IFREMER institute during the EVHOE scientific surveys (October 10th, 2015 [2]). From these individuals, nine tissues (brain, eye, heart, kidney, liver, muscle, ovaries, skin, and testes) were dissected onboard, immediately immersed in RNAlater (Invitrogen), and stored at −20 °C until further processing.

### 2.2 RNA extraction, library construction, and sequencing

Total RNA from nine tissues (Table 1) and three individuals were extracted using TriZol® Reagent (Life Technologies) and quantified with Agilent 2100 Bioanalyzer combined with Agilent RNA 6000 Nano chips (Agilent Technologies, Inc.) at the Gene Expression Unit (SGIker) of the University of the Basque Country UPV/EHU. Samples with RNA integrity numbers (RIN) below 8 were immediately discarded. For every tissue, the sample with the highest RIN was used for sequencing. The exception was testes since there was only one male specimen, and ovary, where both samples were used. A multiplex sequencing library was prepared by labeling each sample with specific 10-mer barcoding oligonucleotides. The barcoded RNA-Seq libraries were sequenced using the Illumina HiSeq 2000 platform using one single lane. Sequencing reactions were performed with paired-end 101 bp and strand-specific protocol at the sequencing facility of the CNAG (Center Nacional d'Anàlisi Genòmica, Barcelona, Spain). Base-calling was performed using the Illumina native software.

### 2.3 Read processing, assembly and quality control

Raw reads were processed with Trimmomatic v0.33 [3] using a gentle procedure to remove adapters and low-quality bases, using the parameters 'SLIDINGWINDOW:4:5 LEADING:5 TRAILING:5 MINLEN:25′. The trimmed reads were assembled with Trinity [4], using default parameters with the exception that input reads were single-stranded to optimize the assembly. To understand the reliability of this assembly, a two-fold approach was used to study its completeness and representativeness. First, the transcriptome was analyzed by running BUSCO [5] against the *Actinopterygii* (ray-finned fishes) database. This software compares the transcriptome against a precomputed set of proteins conserved as Single-Copy Orthologs (SCOs) and returns how many of them are found, duplicated, fragmented or missing. Second, the representativeness of reference was obtained with Bowtie2 [6].

### 2.4 Functional annotation and quantification

Functional annotation of the transcriptome was performed with the execution of the protein prediction software TransDecoder v5.0.2 [4] followed by the annotation of both transcripts and proteins with Trinotate v3.0.2 [7].

TransDecoder translated each transcript into the six possible amino acid sequences and filtered out Open Reading Frames shorter than 300 nucleotides. Afterward, each candidate protein was queried against the SwissProt [8] and Pfam-A [9] databases (downloaded on 2018-10-22) and retained those hits with an E-value or domain noise cutoff less than or equal to 1e-5.

Subsequently, Trinotate was executed with default settings and using the same SwissProt and Pfam databases as before, and the same databases and threshold parameters for BLASTX, BLASTP, and hmmscan. Briefly, transcripts, predicted coding-sequences, and proteins are compared against the SwissProt and Pfam databases, and for each positive match, the source sequence inherits the annotation of its entry in its respective database. This way, sequences obtain Gene Ontology [10] and KEGG [11]. Annotations were obtained for 55,781 proteins from at least one database. Fig. 1 shows the Gene Ontology distribution of terms, and the parts of the metabolome covered, according to the KEGG annotation, and generated with the ggplot2 R package [12], and IPath3.0 [13], respectively.

Trimmed reads were pseudo-aligned and quantified with kallisto v0.44.0 [14] and normalized Transcript per Million counts were obtained with Sleuth v0.29.0 [15].

## Ethics Statement

Research complies with the ARRIVE guidelines and was conducted in accordance with the EU directive 2010/63/EU. IFREMER research vessels are under the supervision of the French Ministry of Education and Research. A steering committee evaluates and approves the campaign program.

## Funding Information

We gratefully acknowledge funding from the Basque Government through a predoctoral grant (PRE_2017_2_0169) and from the Basque University System research group IT1233-19, “Applied Genomics and Bioinformatics”. We also acknowledge funding from the IFREMER institute and by FFP (France Filière Pêche) through the project CAPTAIN.

## CRediT Author Statement

**Jorge Langa:** Conceptualization, Methodology, Software, Validation, Formal analysis, Investigation, Resources, Data curation, Writing – original draft, Visualization; **Martin Huret:** Conceptualization, Resources, Writing – review & editing; **Iratxe Montes:** Conceptualization, Investigation, Resources; **Darrell Conklin:** Conceptualization, Writing – review & editing, Supervision; **Andone Estonba:** Conceptualization, Methodology, Supervision, Writing – review & editing, Project administration, Funding acquisition.

## Declaration of Competing Interest

The authors declare that they have no competing financial interests, which could influence the work reported in this article.
